# Accident-related hepatic trauma in a medical clinical center in eastern China: a cross-sectional study

**DOI:** 10.1186/s12893-020-01043-9

**Published:** 2021-01-06

**Authors:** Weidong Hu, Zipeng Xu, Xu Shen, Yanyan Gu, Zhengxing Dai, Jie Chen, Zhenghai Zhu, Ying Zhou, Wanwen Zhao, Chaobo Chen

**Affiliations:** 1 Department of General Surgery, Wuxi Xishan People’s Hospital, Wuxi, No. 1128, Da-Cheng Road, Wuxi, 214105 Jiangsu China; 2 Department of Immunology, Ophthalmology and ORL, Complutense University School of Medicine, 28040 Madrid, Spain

**Keywords:** Hepatic trauma, Damage control surgery, Peri-hepatic packing, Mortality, China

## Abstract

**Background:**

The treatment of hepatic injury can be complex. Medical clinical centers are often the first line hospitals for the diagnosis and treatment of hepatic trauma in China. The aim of the study is to summarize the experience in the diagnosis and treatment of hepatic trauma in one medical clinical center in China.

**Methods:**

This retrospective study included patients with hepatic trauma admitted between January 2002 and December 2019 at the Xishan People’s Hospital of Wuxi. The outcomes were cure rate and death within 14 days post-discharge.

**Results:**

Among the 318 patients with hepatic trauma, 146 patients underwent surgical treatment, and 172 received conservative treatment; three patients were transferred to other hospitals for further treatment; 283 patients were cured, and 35 died. Severe hepatic trauma occurred in 74 patients, with a mortality rate of 31.1% and accounting for 65.7% of total mortality. American Association for the Surgery of Trauma (AAST) grading ≥ III (OR = 3.51, 95%CI: 1.32–9.37, P = 0.012) and multiple organ injury (OR = 7.51, 95%CI: 2.51–22.46, P < 0.001) were independently associated with death. Among patients with AAST grading ≥ III, surgery was an independent protective factor for death (OR = 0.08, 95%CI: 0.01–0.45, P = 0.004). Among patients with ASST ≥ III and who underwent surgery, age (OR = 5.29, 95%CI: 1.37–20.33, P = 0.015) and peri-hepatic packing (PHP) (OR = 5.54, 95%CI: 1.43–21.487, P = 0.013) were independently associated with death.

**Conclusions:**

AAST grading ≥ III and multiple organ injury were independently associated with death. Among patients with AAST grading ≥ III, surgery was an independent protective factor for death. Among patients with ASST ≥ III and who underwent surgery, age and PHP were independently associated with death.

## Background

Liver trauma results from damage to the liver arising from blunt or penetrating abdominal trauma with severity ranging from a minor capsular tear to severe damage to both lobes with associated injury to the portal vein, hepatic vein, or vena cava [1–4]. Hepatic trauma is common and accounts for 16–30% of abdominal trauma [5, 6]. Any blunt or penetrating abdominal trauma is associated with a risk for liver injury, especially if it involves the right side of the abdomen [1–4]. The risk factors include motor vehicle accidents, direct injury from a weapon, punching, gunshots, and stabbings, falls, farming accidents, and industrial accidents [1–4].

In China, alongside the development of modern transportation and the increase of individuals performing high-risk work, the incidence of hepatic trauma has increased on a yearly basis, and the number of emergency cases of hepatic trauma in clinical medical hospitals has also significantly risen [7, 8]. Although, the incidence of traffic and manufacturing accidents is decreasing with the improvement of traffic regulations and complete technology, but there are still a large number of hepatic trauma patients each year due to China's large population base [7]. Previously studies suggested that increasing age alone is an independent risk factor for mortality, even when adjusted for comorbidities [9, 10]. Meanwhile, age is also an indicator of the Emergency Surgery Score (ESS) which was validated recently as an accurate and user-friendly, post-operative mortality risk calculator specific for Emergency General Surgery (ESG) [11]. Unfortunately, the experience in treating hepatic trauma in specialized hepatic surgery in clinical medical hospitals is still limited [4, 12, 13]. Therefore, traumatic hepatic injuries represent a great challenge for general surgeons in clinical medical hospitals, especially those with severe hepatic trauma. Summarizing and sharing practical experiences regarding the treatment of hepatic trauma in clinical medical hospitals will help other surgeons in the treatment of hepatic trauma.

The aim of the present study was to summarize the experience in the diagnosis and treatment of patients with hepatic trauma treated in one hospital between January 2002 and December 2019. The results could help surgeons and policymakers in other clinical hospitals in developing countries.

### Methods

## Patients

This retrospective study included patients with hepatic trauma admitted between January 2002 and December 2019 at the Xishan People’s Hospital of Wuxi. The inclusion criterion was hepatic trauma diagnosis and classified by the American Association for the Surgery of Trauma (AAST) [14]. The exclusion criterion was patients who died before arriving at the emergency room or during emergency room rescue.

The patients were stratified by AAST grading and divided into the surgery and non-surgery groups. The patients in the surgery group were divided into the peri-hepatic packing (PHP) surgery and definite surgery groups. This study was approved by the ethics committee of Wuxi Xishan People’s Hospital. The requirement for informed consent was waived.

### Diagnosis and treatments

All patients were diagnosed as soon as possible according to the AAST guidelines [14]. For patients with unstable vital signs at admission, frequent movements and unnecessary examinations were avoided as much as possible. The preliminary diagnosis of suspected severe hepatic trauma could be made according to the history of right quarter costal and thoracodorsal traumas and clinical manifestations including right upper abdominal pain, ecchymosis, scratches, tenderness, rebound pain, and hemorrhagic shock, while only some simple and rapid examinations such as diagnostic abdominal puncture and bedside ultrasound were allowed. Abdominal CT was performed for patients with suspected hepatic trauma whose hemodynamics were stable or blood pressure could be maintained at a normal level by slight fluid replacement. This was not only helpful for a definite diagnosis, determination of the severity of the hepatic injury, dynamic assessment of injury changes and prognosis, but it could also provide help for the early detection of injury to other abdominal organs, including combined injury of the spleen, hollow viscus, and retroperitoneal organs.

#### Surgery

All the patients in the operation group were completed by the doctors in the same operation group according to the enrolled patients' surgical indications previously published [15–17].

The principle of surgical treatment for hepatic trauma is to determine the traumatic condition, debride thoroughly, accurately stop bleeding, eliminate bile leakage, and establish unobstructed drainage time-limited [18]. Once the traumatic condition meets the surgical indications, it is necessary to seize the opportunity of surgical exploration and follow the principle of damage control (including the control of primary injury and secondary injury caused by surgery). Currently, it is widely accepted that the principle of damage control surgery should be followed when rescuing patients with severe hepatic trauma with unstable hemodynamics [19]. If the surgeons directly expose and suture the hepatic fissure regardless of the unstable vital signs of the wounded, it may aggravate bleeding and shock, and induce the lethal triad manifested by severe acidosis, hypothermia, and coagulation dysfunction, possibly leading to even cardiac arrest and the loss of surgical opportunities. If blood pressure was lower than 80/50 mmHg (1 mmHg = 0.133 kPa) and showed a progressive decline during surgery, anatomy restoration and suture were suspended. PHP temporarily performed using a dry gauze pad was suggested, assisted with compression using hands for hemostasis and effective liquid resuscitation (i.e., plasma-based liquid resuscitation with a limited amount of crystalloid) [17].

The indications of PHP were: (1) hemorrhage was too important for the wounded to tolerate complicated surgery; (2) severe hepatic trauma accompanied by massive hemorrhage, coagulation dysfunction, and extensive bleeding in the wound after massive blood transfusion; (3) the bleeding site was difficult to be exposed, and other methods were ineffective for hemostasis, such as the hepatic laceration extending to the first hepatic hilum via the subhepatic approach which was difficult to be exposed, hemostatic suture and extensive subcapsular hematoma that was invalid and still expanding after hepatic artery ligation; (4) blood supply was lacking, or technical conditions were limited, and the patients received temporary hemostasis and were transferred to another hospital for further treatment [13, 20]. Further surgery was performed until the patients’ blood pressure could be maintained at about 90/60 mmHg, or the central venous pressure was slightly higher than the normal level (to avoid hyperpiesia or hypotension).

As for the surgical methods, superficial lacerations < 3 cm could be directly sutured by the horizontal mattress or “8” suture through the bottom of the wound. For regular deep lacerations > 3 cm, the finger fracture technique could be used to remove inactivated liver and rapidly ligate deep wounds or suture various pipeline structures, and then hepatic fissures could be sutured with packing the greater omentum combined with bottom-up suture. Debridement hepatectomy was feasible for an irregular stellate or comminuted hepatic laceration. PHP could be used to treat severe and complex hepatic injury involving both left and right hepatic lobes and even main hepatic veins and retro hepatic inferior vena cava whose bleeding was difficult to control. Long-term regular hepatectomy was avoided unless necessary. Simultaneously, no matter what surgery method was chosen, the exact hemostasis should be achieved simply, effectively, and quickly [13].

#### Conservative treatments

It is generally believed that AAST grade III or below hepatic injury belongs to mild hepatic injury, which can be treated conservatively [21]. Mainly, the patients were absolutely advised to stay in bed and avoid strenuous activities. The changes in vital signs, hemodynamics, and erythrocyte specific volume were closely observed. The patients were instructed to fast and to achieve gastrointestinal decompression, while the fluid diet was given until anal ventilation. The conservative treatments included using hemostatic drugs to stop bleeding, antibiotics to prevent abdominal infection, and fluid supplementation to maintain the vital signs. The indications for conservative treatments of hepatic injury included: (1) closed hepatic trauma with stable hemodynamics or stable hemodynamics after fluid therapy was stable or improved by dynamic CT examination; and (2) splenic, hollow viscus, pancreatic, renal, and other abdominal or retroperitoneal organ injuries requiring surgical treatment were excluded. Ultrasound and/or CT were reviewed in time according to the demand of the patients.

### Data collection and outcomes

Data collected included: age, sex, causes of injury, AAST classification of the trauma (Including open injury and closed injury), combined injury, surgery or not, accurate repair of liver injury to stop bleeding, and PHP treatment. The age was converted into a dichotomous variable according to the median.

The outcomes were cure rate and death within 14 days post-discharge. Cured was defined as a patient having no symptoms of abdominal pain or fever, and no obvious abnormality was detected by CT, ultrasound, and hematological examination.

### Statistical analysis

The data were analyzed using SPSS 22.0 (IBM, Armonk, NY, USA). The continuous data were expressed as means ± standard deviations and analyzed using Student’s t-test. Categorical data were presented as frequencies and percentages and were analyzed using Fisher’s exact test. Univariable logistic regression was used to analyze the factors associated with death. Variables with P < 0.05 in the univariable analyses and concerned in the study were included in a multivariable logistic regression (enter method). P value < 0.05 were considered statistically significant.

## Results

### Characteristics of the patients

A total of 318 patients with hepatic trauma were included in the study. Thirty-five (11.0%) patients died, and 283 (89.0%) patients were successfully rescued and cured. Among the 318 patients, 25 cases were caused by open injury, 293 by closed injury, 201 by traffic accidents, 68 by production safety accidents, 38 by personal injury, and 11 by other accidents. Among the 318 patients, 146 patients were treated surgically, and 172 were treated non-surgically. The general data of the two groups are detailed in Table 1.

**Table 1:** Characteristic and clinical features of patients

	Total (n = 318)	AAST grading I-II			AAST grading III or above		
		Non-surgery (n = 163)	Surgery (n = 81)	P	Non-surgery (n = 9)	Surgery (n = 65)	P
Male, n (%)	189 (59.4)	96 (61.0)	30 (37.0)	**0.001**	9 (100)	54 (83.1)	0.402
Age, year, mean ± SD	47.2 ± 11.5	47.4 ± 11.8	47.1 ± 11.3	0.850	47.5 ± 6.2	47.4 ± 12.7	0.943
Age, n (%)				**0.012**			0.360
≤ 52	154	65	46		7	36	
> 52	164	98	35		2	29	
Causes of injury, n (%)				** < 0.001**			
Traffic accidents	201 (63.2)	91 (55.8)	68 (84.0)		5 (55.6)	37 (57.0)	0.715
Production safety accidents	68 (21.4)	42 (25.8)	6 (7.4)		2 (22.2)	18 (27.7)	
Personal injury	38 (11.9)	23 (14.1)	6 (7.4)		1 (11.1)	8 (12.3)	
Other accidents	11 (3.5)	7 (4.3)	1 (1.2)		1 (11.1)	2 (3.1)	
Close injury, n (%)	301 (94.7)	162 (99.4)	70 (86.4)		7 (77.8)	62 (95.4)	0.109
Combined with other organ injuries, n (%)	97 (30.5)	20 (12.3)	21 (25.9)	** < 0.001**	6 (66.7)	50 (77.0)	0.797
PHP, n (%)	19 (6.0)	0	1 (1.2)		0	18 (27.7)	0.161
Death, n (%)	35 (11.0)	4 (2.5)	8 (9.9)	**0.027**	7 (77.8)	16 (24.6)	**0.004**

Bold values indicate a P-value less than 0.05 is statistically significant

*AAST* American Association for the Surgery of Trauma, *SD* standard deviation, *PHP* peri-hepatic packing

Among the 318 patients, 146 were treated surgically, and 172 were treated non-surgically. The surgical methods in the surgery group included simple hepatic repair and suture (n = 112), partial hepatectomy (n = 6), hepatic repair + partial hepatectomy (n = 7), hepatic repair + PHP (n = 10), hepatic repair + repair of the right hepatic vein and inferior vena cava + PHP (n = 8), and partial hepatectomy + repair of the portal vein and inferior vena cava + PHP (n = 3). Contusion and laceration of the liver were found in another 25 patients after reaching the abdomen, and bleeding was stopped successfully. Hemostasis was successfully achieved in one patient only by electrocoagulation. In the surgery group, 19 patients were combined with splenectomy, two with splenic repair, three with nephrectomy, nine with diaphragm repair, two with splenectomy + gastric repair, one with splenectomy and distal pancreatectomy, three with the repair of the stomach and duodenum bulb, one with small intestinal repair, three with small intestinal mesenteric repair, one with partial resection of the transverse colon, and two with splenectomy + partial resection of the duodenum and small intestine.

A total of 74 patients with severe hepatic trauma were classified as AAST grade ≥ III; 23 patients died directly of severe hepatic trauma, with a mortality rate of 31.1% in severe hepatic trauma patients, or 7.2% among all patients. There were 244 patients with hepatic trauma below grade III, but none of whom died directly from hepatic trauma. Among the patients with AAST grade I-II, compared with the non-surgery group, the percentage of males was lower in the surgical group, the frequency of traffic accidents was higher, the frequency of multiple organ injury was higher, and the occurrence of death was higher (all P < 0.05). Among the patients with AAST grade ≥ III, compared with the non-surgery group, the occurrence of death was lower in the surgery group (P = 0.004).

### Univariable and multivariable analysis for death in all patients

The multivariable analysis showed that AAST grading ≥ III (OR = 3.51, 95%CI: 1.32–9.37, P = 0.012) and multiple organ injury (OR = 7.51, 95%CI: 2.51–22.46, P < 0.001) were independently associated with death in all patients (Table 2).

**Table 2:** Univariable and multivariable analyses for death in all patients

Item	Univariable			Multivariable		
	OR	95%CI	P	OR	95%CI	P
Male	6.13	2.11–17.83	**0.001**	1.93	0.57–6.46	0.289
Age > 52 years	0.99	0.49–2.01	0.986			
AAST grading >III vs grading I-II	8.72	4.07–18.66	**< 0.001**	3.52	1.32–9.37	**0.012**
Open injury vs close injury	1.8	0.49–6.61	0.375			
Combined with other organ injuries	15.28	6.09–38.36	**< 0.001**	7.51	2.51–22.46	**< 0.001**
Surgery	2.88	1.36–6.10	**0.006**	0.72	0.26–2.01	0.535

Bold values indicate a P-value less than 0.05 is statistically significant

*OR* odds ratio, *CI* confidence interval, *AAST* American Association for the Surgery of Trauma

### Univariable and multivariable analyses for death in patients with AAST grading ≥ III

Among patients with AAST grading ≥ III, surgery was an independent protective factor for death (OR = 0.08, 95%CI: 0.01–0.45, P = 0.004) (Table 3).

**Table 3:** Univariable and multivariable analyses for death in patients with AAST grading ≥ III

	Univariable			Multivariable		
	OR	95%CI	P	OR	95%CI	P
Male	1.24	0.30–5.18	0.768			
Age > 52 years	1.84	0.6 8–4.97	0.231			
Close injury	0.59	0.06–5.06	0.585			
Combined with other organ injuries	1.80	0.52–6.22	0.355	2.52	0.60–10.65	0.208
Surgery	0.09	0.02–0.50	**0.005**	0.08	0.01–0.45	**0.004**

Bold values indicate a P-value less than 0.05 is statistically significant

*OR* odds ratio, *CI* confidence interval

### Clinical features of PHP surgery and definite surgery subgroups

Table 4 presents the characteristics of the patients with AAST grading ≥ III. The frequency of males was higher in the definitive surgery group compared with the PHP group (P = 0.011). Otherwise, the other characteristics were similar.

**Table 4:** Clinical features of PHP surgery and definitive surgery subgroups (AAST grading ≥ III)

	Definitive surgery group (n = 47)	PHP surgery group (n = 18)	P
Male, n (%)	43 (91.5)	11 (61.1)	**0.011**
Age, year, mean ± SD	46.9 ± 11.7	48.4 ± 8.5	0.622
Closed n (%)	44 (93.6)	18 (100)	0.555
Combined with other organ injuries, n (%)	36 (76.6)	14 (77.8)	0.820
Cured/died	39/8	11/8	0.123

Bold value indicate a P-value less than 0.05 is statistically significant

*AAST* American Association for the Surgery of Trauma, *SD* standard deviation, *PHP* peri-hepatic packing

Of the 146 patients who underwent surgery, 122 were cured, and 24 died. Among them, eight died of combined severe craniocerebral or thoracic trauma and 16 of hepatic rupture above grade III. In the surgery group, 19 patients with hepatic rupture underwent PHP because of severe liver damage and uncontrollable bleeding (P < 0.05). Among them, eight patients died from uncontrollable bleeding during surgery or within two days after surgery, and six patients obtained successful hemostasis by intraoperative packing.

Among the eight dead patients, one died of combined brain contusion and laceration accompanied by hemorrhage, brain swelling, diffuse axonal injury, and eventual rescue failure. One patient was transferred to the General Hospital of Nanjing Military Region, another medical clinical center located in Nanjing, on the 9th day after surgery at the request of family members to remove the packing materials. Bile leakage and repeated subphrenic infection occurred after surgery. Fortunately, with active anti-infection and supportive treatment, the patient recovered. One patient was transferred to Wuxi People’s Hospital on the 5th day after surgery at the request of his family and was finally cured with continuing efforts. One patient was complicated with abdominal compartment syndrome and renal failure 3 days after surgery and was finally cured and discharged after bedside continuous venovenous hemofiltration (CVVH) and abdominal decompression (with incision and temporary closure of the abdominal cavity using artificial film). One patient was treated with removal of the packing materials for several times from the 6th day after surgery, and finally recovered and discharged. One patient was discharged initiatively on the 5th day after surgery and failed to remove the packing materials.

### Associated factors with death in surgery group with ASST III or above

Among patients with ASST ≥ III and who underwent surgery, age (OR = 5.29, 95%CI: 1.37–20.33, P = 0.015) and PHP (OR = 5.54, 95%CI: 1.43–21.487, P = 0.013) were independently associated with death (Table 5).

**Table 5:** Factors associated with death in the surgery group with ASST ≥ III

	Univariable			Multivariable		
	OR	95%CI	P	OR	95%CI	P
Male	0.83	0.19–3.57	0.797			
Age > 52 years	3.91	1.17–13.05	**0.026**	5.29	1.38–20.325	**0.015**
Combined with other organ injuries	1.52	0.37–6.21	0.558			
PHP	4.00	1.21–13.28	**0.024**	5.54	1.43–21.487	**0.013**

Bold values indicate a P-value less than 0.05 is statistically significant

*OR* odds ratio, *CI* confidence interval, *PHP* peri-hepatic packing

### Characteristics of the non-surgery group

Among the 161 patients in the non-surgery group, one was transferred to Wuxi People’s Hospital at the request of the family after stable conditions with short-term conservative treatment. Eleven patients died, among which seven died of hepatic rupture above grade III, and two of them were combined severe craniocerebral and thoracic trauma. All of them died.

## Discussion

The treatment of hepatic injury can be complicated. Unfortunately, the expertise level in specialized hepatic surgery in clinical medical hospitals is generally low. This study aimed to summarize the experience in the diagnosis and treatment of hepatic trauma in one clinical hospital in China. The results suggest that despite improvements in technologies for liver surgery, the level of hepatic trauma repair in clinical medical hospital is low partly because of the limitations in experience and hospital conditions, while most important is because of emergency incident to hepatic trauma itself. AAST grading ≥ III and multiple organ injury were independently associated with death. Among patients with AAST grading ≥ III, surgery was an independent protective factor for death. Among patients with ASST ≥ III and who underwent surgery, age and PHP were independently associated with death.

Over the past 20 years, with the continuous accumulation of experience in the treatment of severe hepatic trauma, the establishment of novel concepts for trauma treatment and the improvement in treatment methods, the mortality of grade III and IV hepatic trauma has dropped to less than 10% in large hospitals [13]. On the other hand, the data from clinical medical centers are not optimistic: in the past 15 years, the mortality of severe hepatic trauma in the hospital reached 25.4%, and none of the patients below grade III died of hepatic trauma directly [14]. Therefore, for clinical medical hospitals, it is particularly necessary to attach great importance to the treatment of severe hepatic trauma above grade III and strive to improve the success rate.

First, for patients with severe hepatic injury, time means life. During the rescue, attention should be paid to every detail and factors that may delay diagnosis and treatment. As far as possible, emergency trauma rescue teams should be set up in clinical medical hospitals. Once severe trauma occurs, ambulance first-aid personnel can inform the emergency department of hospitals to open the fast-track channel and arrange the rescue team members and various bedside examination machines. The blood transfusion department can inform the central blood bank to prepare a large amount of blood. For patients with suspected severe hepatic injury, venous access is preferred to the upper limb vein, internal jugular vein, or subclavian vein. The superficial veins collapse and are difficult to puncture, and repeated puncture should be avoided to save time. The internal jugular vein or subclavian vein should be catheterized by an experienced anesthesiologist. For patients with suspected severe hepatic injury and unstable vital signs, liquid resuscitation should be carried out as soon as possible according to the principle of crystalloid fluid first and then colloid fluid (crystal:colloid ratio of 2–3:1) before the blood supply arrives, so as to maintain blood pressure, improve shock, prevent cardiac arrest, and gain valuable time for further surgery to stop bleeding. Four patients with severe hepatic injury in this study were delayed for a long time due to various causes at the scene of the accident. Although they were rescued with the best efforts after being sent to the hospital, they still died of hemorrhagic shock and multiple organ failure before laparotomy due to excessive intraperitoneal bleeding, which exceeded the patients’ physiological compensation limit. Therefore, the length of time after injury is an extremely important factor for whether or not patients with a severe hepatic injury can be successfully rescued.

The time from injury is very important for wounded patients with suspected abdominal bleeding, especially with short injury time. Bedside ultrasound and intensive monitoring should be performed first to determine the amount of abdominal bleeding and observe the stability of the vital signs. The temporary “stability” of the initial vital signs will lead to increased bleeding, shock, and even death during the examinations. Simultaneously, negative ultrasound results cannot completely exclude liver damage. Nowadays, abdominal CT is considered to be the preferred method for the diagnosis of abdominal injury in patients with stable hemodynamics [22]. After the diagnosis of hepatic injury, injury assessment is necessary to select a reasonable treatment strategy according to the grade of hepatic injury, according to AAST [15, 23, 24]. Although CT and ultrasound are important methods for the diagnosis of hepatic trauma, they cannot accurately reflect the AAST grade. The velocity and amount of abdominal hemorrhage and the stability of circulation are the most direct indicators of the severity of hepatic injury [13, 22, 25].

The indications for non-surgical and surgical treatments should also be understood, especially in the presence of grade III or above injury [21]. Emphasizing the importance of non-surgical treatment is a major change in the concept of treating hepatic trauma over the last 20 years [26], and nowadays, non-surgical treatment for severe hepatic trauma has become a tendency [27, 28]. Namely, the determinants of non-surgical treatment for hepatic trauma lie in whether the hemodynamics of patients are stable, and whether there are other combined injuries requiring surgical treatment, rather than relying unilaterally on the grading of hepatic trauma and intraperitoneal blood accumulations. In this study, although 14 patients presented large intraperitoneal blood accumulations, it was found that the bleeding at the hepatic laceration stopped spontaneously during laparotomy. It should be noted that the conservative treatment for hepatic trauma combined with splenic or renal contusion and laceration should be very careful. For such patients, close attention should be paid to the changes in vital signs and abdominal signs under intensive monitoring. Ultrasonography or CT should be reviewed timely, and delayed splenic or renal rupture should be monitored, which is the most common cause of the failure in conservative treatment for hepatic trauma. Based on many years of clinical experience, the authors’ opinion is that although non-surgical treatment has gradually turned into the main treatment for hepatic trauma, conservative treatment for severe hepatic trauma above grade III still needs to be carefully selected. Especially in clinical medical hospitals, good monitoring conditions and experienced team of liver surgery are missing. Once conservative treatment fails, the rapid and effective surgical transfer cannot be ensured, and surgical indications should be expanded.

When doing an operation, PHP is a very important and practical technique in damage control surgery (DCS) [29]. Inferior vena cava and hepatic vein are low-pressure systems. The effect of PHP on such venous hemorrhage is efficacious [20, 30, 31]. Sometimes, it is difficult to find the exact bleeding site during surgery, and the Pringle maneuver can be used to block hepatic blood flow from the first porta hepatis. During the operation, when there is still a large amount of dark red blood gushing from the hepatic fissure, it should be considered that the bleeding originates from the hepatic vein, short hepatic vein, and/or retro-hepatic inferior vena cava laceration. Such injuries are particularly difficult for general surgeons to treat in clinical medical hospitals. In addition, catastrophic hemorrhage is often caused by dissecting, exposing, and suturing the bleeding site. Extensive and uncontrollable bleeding during the surgical incision, abdominal cavity, and hepatic wound indicates that the body has severe coagulation dysfunction, and the patient is on the verge of death and may be unable to tolerate further surgery. At this moment, surgery should be completed after effective PHP using dry gauze pad (gelatin sponge or omentum can be used between the gauze pad and the liver surface to prevent secondary bleeding when removing the gauze). Actually, accurate and skillful application of this technology is related to the success of saving patients’ lives and can gain time for emergency transfer to superior hospitals with better technical conditions for further rescue. In addition, the significance of PHP is also that, when hepatic rupture is intraoperatively found to be combined with splenic rupture, mesenteric laceration and simultaneous massive hemorrhage of multiple organs in the abdominal cavity, temporary PHP can be used to control bleeding at the site of hepatic injury, and then other bleeding foci such as splenic rupture and mesenteric rupture can be treated calmly. According to our experience, the surgeons determine patients’ injury and physiological state in advance and try to perform PHP actively and decisively before patients’ general condition deteriorates, instead of being forced to perform PHP in a hurry when patients show physical exhaustion and severe coagulation dysfunction. Second, excessive gauze packing can compress the inferior vena cava and renal vein, which might lead to abdominal compartment syndrome and might aggravate hepatic laceration and hemorrhage. The packing can be removed 72 h to one week after surgery, depending on the condition of patients. Early removal may lead to re-bleeding, and late removal may cause an increased risk of abdominal infection [32]. In order to reduce the incidence of abdominal infection after surgery for severe hepatic rupture, effective drainage was placed around the liver (subphrenic, subhepatic and hepatic section) and pelvic cavity during PHP in the first surgery and packing removal in the second surgery, and timely dressing change could be made to prevent retrograde infection.

This study has limitations. It was a single-center study with retrospectively analysis. There was no comparator group from higher-level hospitals.

## Conclusion

This study summarized the experience in the diagnosis and treatment of hepatic trauma in a clinical medical hospital. Despite improvements in technologies for liver surgery, the level of hepatic trauma repair in clinical medical hospitals is low because of the limitations in experience and hospital conditions. AAST grading ≥ III and multiple organ injury were independently associated with death. Among patients with AAST grading ≥ III, surgery was an independent protective factor for death. Among patients with ASST ≥ III and who underwent surgery, age and PHP were independently associated with death.

## Data Availability

The datasets used and/or analyzed during the current study available from the corresponding author on reasonable request.

## Abbreviations

AAST: American Association for the Surgery of Trauma
CVVH: Continuous venovenous hemofiltration
DCS: Damage control surgery
DIC: Disseminated intravascular coagulation
ESS: Emergency surgery score
ESG: Emergency general surgery
PHP: Peri-hepatic packing
